# Behavioral phenotype in five individuals with *de novo* mutations within the *GRIN2B* gene

**DOI:** 10.1186/1744-9081-9-20

**Published:** 2013-05-29

**Authors:** Inga Freunscht, Bernt Popp, Rainer Blank, Sabine Endele, Ute Moog, Holger Petri, Eva-Christina Prott, Andre Reis, Jochen Rübo, Bernhard Zabel, Martin Zenker, Johannes Hebebrand, Dagmar Wieczorek

**Affiliations:** 1 Department of Child and Adolescent Psychiatry, Psychosomatics and Psychotherapy, University of Duisburg-Essen, Essen 45147, Germany; 2 Institute of Human Genetics, University of Erlangen-Nuremberg, Erlangen, Germany; 3 Klinik für Kinderneurologie und Sozialpädiatrie, Kinderzentrum Maulbronn gGmbH, Maulbronn, Germany; 4 Institute of Human Genetics, University of Heidelberg, Heidelberg, Germany; 5 DRK-Kinderklinik, Siegen, Germany; 6 Institut für Humangenetik, University of Duisburg-Essen, University Hospital Essen, Essen, Germany; 7 Klinik für Kinder- und Jugendmedizin, St. Antonius-Hospital Kleve, Kleve, Germany; 8 Centre for Pediatric and Adolescent Medicine, University Hospital Freiburg, Freiburg, Germany; 9 Institut für Humangenetik, Otto-von-Guericke University Magdeburg, Magdeburg, Germany

**Keywords:** *GRIN2B* mutations, Behavior problems, Hyperactivity, Stereotypies, Intellectual disability

## Abstract

**Background:**

Intellectual disability (ID) is often associated with behavioral problems or disorders. Mutations in the *GRIN2B* gene (MRD6, MIM613970) have been identified as a common cause of ID (prevalence of 0.5 – 1% in individuals with ID) associated with EEG and behavioral problems.

**Methods:**

We assessed five *GRIN2B* mutation carriers aged between 3 and 14 years clinically and via standardized questionnaires to delineate a detailed behavioral phenotype. Parents and teachers rated problem behavior of their affected children by completing the Developmental Behavior Checklist (DBC) and the Conners’ Rating Scales Revised (CRS-R:L).

**Results:**

All individuals had mild to severe ID and needed guidance in daily routine. They showed characteristic behavior problems with prominent hyperactivity, impulsivity, distractibility and a short attention span. Stereotypies, sleeping problems and a friendly but boundless social behavior were commonly reported.

**Conclusion:**

Our observations provide an initial delineation of the behavioral phenotype of *GRIN2B* mutation carriers.

## Background

Intellectual disability (ID) is defined as impairment of cognitive and adaptive functions and occurs with an incidence of about 2% in the general population [1]. It is suspected that the involvement of many genes will be discovered during the upcoming years via identification of novel mutations with major effect sizes. Several recently published papers demonstrate that exome sequencing is a powerful tool for the identification of the genetic basis of ID [2,3]. One of these papers shows that *de novo* point mutations and small indels account for up to 45-55% of patients with severe ID with high locus heterogeneity [2], so it can be assumed that the number of gene mutations involved in ID is higher than previously expected.

Early diagnosis of ID leads to improvement of psychoeducation, genetic counseling of the families and potentially of therapy in the affected individuals. This is important, because children and youths with ID are at high risk for various behavioral problems and psychiatric disorders [4-6]. Recently, mutations in the *GRIN2A* and *GRIN2B* genes, which encode for subunits of N-methyl-D-aspartate (NMDA) receptors, have been identified in individuals with ID, EEG anomalies/seizures and behavioral anomalies [7]. NMDA receptors are widely expressed in the central nervous system. They form the major molecular determinants of excitatory synapses [8], and they are implicated in learning and memory [9].

The prevalence of *GRIN2B* mutations in intellectually disabled children has been estimated to be 0.5-1% [7]. The behavioral phenotype has not yet been described in greater detail; investigators have merely pointed out that the respective children show behavioral anomalies. In 2011, a single individual with a *GRIN2B* mutation was identified within a group of 20 individuals with autism spectrum disorders [10]. The girl had a full scale IQ of 63, met the criteria for autistic disorder and showed hyperactivity., Three further *de novo* mutations in *GRIN2B*, all predicted to be protein-truncating, were reported very recently in a dataset of 2,500 individuals (1,703 simplex ASD probands and 744 controls), but no detailed clinical data are available [11]. Genetic variations in NMDA receptors have been implicated in the genetic susceptibility to neurological, psychiatric and learning disorders, e.g. obsessive-compulsive disorder [12], attention deficit/hyperactivity disorder (ADHD) [13], dyslexia [14], schizophrenia and bipolar disorders [15], Parkinson disease and Huntington disease [16]. It is still an open debate whether patients with *GRIN2B* mutations present with an unspecific ID phenotype with some anomalies in behavior, or with a very specific behavioral phenotype that could lead to the suspicion of a *GRIN2B* mutation on clinical evidence alone. The latter would facilitate making the diagnosis and improve genetic counseling in the families.

Four of the individuals reported here were previously published by us (subjects 1, 2, 5 and 9 within Endele et al., 2010 [7]) with a focus on the identification of the causative genetic defect. At that time, the clinical data published were limited. All other reports dealing with *GRIN2B* mutations also focused on other aspects [7,10-14,17]. Meanwhile, we have identified an additional, previously undescribed female. For the first time, we provide a detailed clinical synopsis of the behavioral phenotype associated with *GRIN2B* mutations.

## Individuals and methods

### Affected individuals

**Figure 1:**
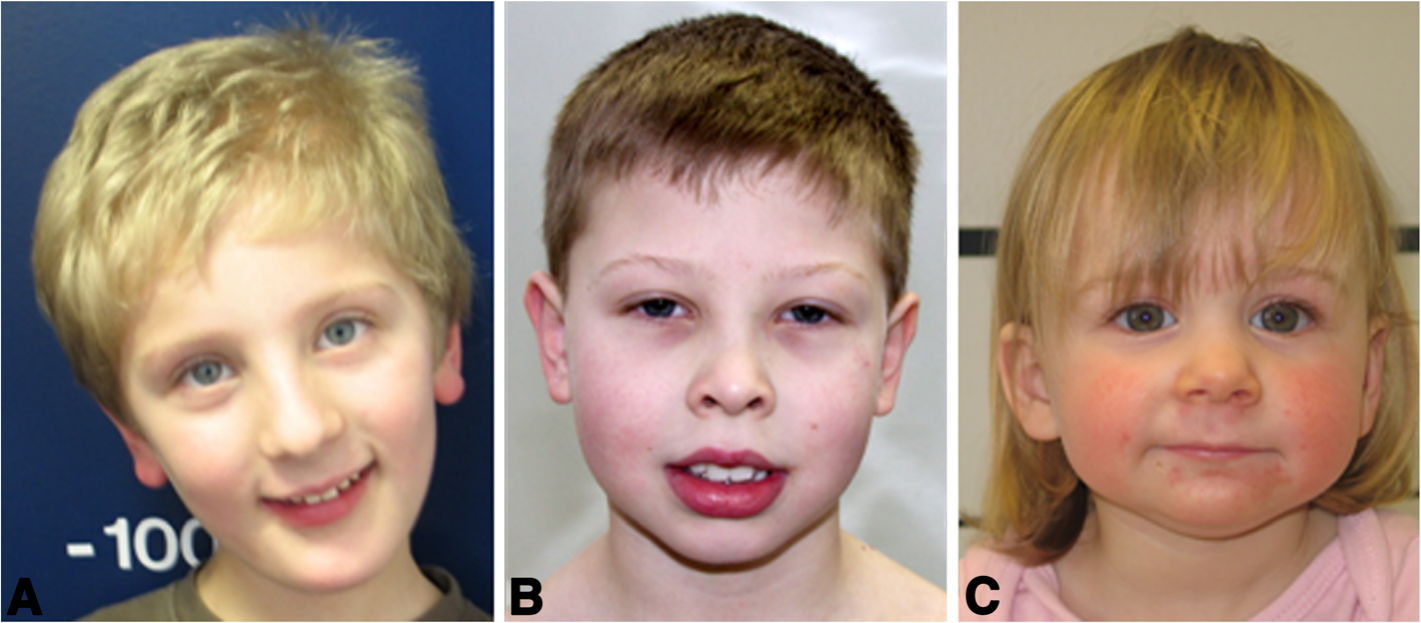
**Unremarkable facial phenotypes in individuals with *GRIN2B *mutations. A**. Individual 1 at the age of 5 years. **B**. Individual 3 at the age of 8 years. **C**. Individual 5 at the age of 2 years.

The mean age of the 5 children (2 females, 3 males) at the last physical examination was 9 years, ranging from 23 months to 13 years. Individuals were identified within the study of Endele et al., 2010 [7], and through sequencing of an additional cohort of 93 individuals with ID collected within the German Mental Retardation Network. Affected individuals and their parents were assessed within the different departments of human genetics, pediatric hospitals or departments of child and adolescent psychiatry. We obtained written informed consent from the families of the index patients for participation in this study. The study was performed according to the Declaration of Helsinki protocols and was approved by the local institutional review board (ethical votum 08-3663 for MRNET). All parents gave written consent to publish the data including the photographs (Figure 1). The clinical data are summarized in Table 1.

**Table 1:** **Clinical data in individuals with*de novo GRIN2B*mutations**

	**Individual 1 (subject 1, Endele et al., 2010)**	**Individual 2 (subject 2, Endele et al., 2010)**	**Individual 3 (subject 5, Endele et al., 2010)**	**Individual 4 (subject 9, Endele et al., 2010)**	**Individual 5 (this report)**
**Mutation**	Translocation with breakpoint in *GRIN2B*	Translocation with breakpoint in *GRIN2B*	c.2044C > T (p.R682C)	c.803_804delCA (p.T268SfsX15)	c.1906G > C (p.A636P)
**Ethnic origin**	German	German	German	German	German
**Sex**	Male	Male	Male	Female	Female
**Gestational weeks at birth**	41	38	40	39	40
**Birth weight [g(SD)]**	3535 (mean)	3390 (mean)	4000 (0.9)	3720 (0.9)	3940 (1.1)
**Birth length [cm(SD)]**	52 (−0.4)	52 (mean)	54 (−0.7)	55 (1.8)	53 (0.6)
**OFC at birth [cm(SD)]**	34 (−1.6)	32.5 (−2.1)	34 (−1.2)	34 (−0.5)	not reported
**Age at last physical examination [years]**	5 ^3^/_12_	12	13	13	1 ^11^/_12_
**Age at behavioral assessment**	6 ^8^/_12_	14	14 ^6^/_12_	14 ^8^/_12_	3 ^10^/_12_
**Height [cm(SD)]**	123 (1.8)	150 (mean)	154 (−0.1)	150 (−1.0)	87 (−0.2)
**Weight [kg(SD)]**	23 (1.8)	36 (−1.5)	53 (0.8)	41 (2.0)	10 (−2.3)
**OFC [cm(SD)]**	50 (−1.0)	50 (−2.4)	54 (−0.9)	54.5 (0.2)	47 (−0.8)
**Intellectual disability**	Mild	Severe	Mild	Moderate	Mild
**Walking age [months]**	23	36	20	24	25
**First words [months]**	12	-	18	28	10
**Seizures**	-	-	-	-	-
**EEG**	Left-sided sharp wave complexes	Slow dysrhythmia, occipital abortive sharp waves	Normal	Normal	Sharp wave complexes temporoparietal
**Cranial MRI**	Normal	Hydrocephalus externus	Normal	Not performed	Normal
**Behavior:**					
**-hyperactivity**	+	+	/	+	+
**-short attention span**	+	+	+	+	+
**-sleep disturbance**	+	+	/	+	+
**-aggressiveness**	+	+	+	/	-
**-stereotypies**	+	+	+	+	-
**-friendliness**	+	+	+	+	+
**Others**	/	Cryptorchidism, choanal atresia, inguinal hernia	/	/	/

### Behavioral assessments

Behavioral problems were measured by ratings of parents and teachers for all five mutation carriers. The behavioral data presented in the case reports are based on parental reports and clinical observation. From February to December 2011, questionnaires were sent to the families and filled out at home. The teachers were contacted by the parents and concomitantly filled in the questionnaires. The individuals’ ages at behavioral assessment ranged from 3 ^10^/_12_ to 14 ^8^/_12_ years. The Developmental Behavior Checklist (DBC) and the Conners’ Rating Scales-Revised (CRS-R) questionnaires, described in detail in the following section, were used by parents and teachers to assess the behavioral problems.

### Developmental behavior checklist (DBC)

We used the German version [18] of the Developmental Behavior Checklist (DBC) [19] to assess behavioral and emotional problems in the five mutation carriers with ID (designed for children aged 4 to 18). The version for primary caregivers/parents, termed DBC-P, includes 96 items that are rated on a three-point scale ranging from 0 (not true) to 1 (sometimes/somewhat true) to 2 (often/very true). The items allow calculation of Total Problem Behavior Score and classification into one of five subscales derived from factor analysis: Disruptive, Self-Absorbed, Communication Disturbance, Anxiety and Social Relating. The version for teachers, DBC-T, contains 94 items with the same scales. Raw scores of the five subscales and the Total Problem Behavior Score can be transformed into standardized percentile ranks based on reference populations comprised of intellectually disabled children. Scores higher than 84 (> 1 Standard Deviation (SD) from mean) are termed as clinically relevant.

The German version of the DBC-P shows satisfying psychometric properties in internal consistency, retest reliability and discriminant validity [20]. Standardization of the parent’s version was based on 721 German children and adolescents with ID [21]. For the DBC-T, an Australian analysis revealed satisfying psychometric properties [19,22]. Because of lack of German norms for the teacher’s version, we used the Australian DBC-T standards.

### Conners’ rating scales-revised (CRS-R)

The CRS-R [23] assesses symptoms of attention deficit/hyperactivity disorder (ADHD) and related behavioral problems in children and adolescents (aged 3 to 17). Long and short versions are available for ratings by parents, teachers and for adolescent self-report. We used the long German versions for parents (CPRS-R:L) and teachers (CTRS-R:L). The CPRS-R:L and CTRS:L contain 80 and 59 items, respectively, that are rated on a four-point scale with 0 = not true at all (never, seldom), 1 = just a little true (occasionally), 2 = pretty much true (often, quite a bit) and 3 = very much true (very often, very frequent). Both versions provide the following subscales: Oppositional, Cognitive Problems/Inattention, Hyperactivity, Anxious-Shy, Perfectionism, Social Problems, Conners’ ADHD-Index, Conners’ Global Index (Restless-Impulsive, Emotional Lability and Total) and DSM-IV Symptom Subscales (Inattentive, Hyperactive-Impulsive and Total). The Psychosomatic subscale is only present in the CPRS-R:L questionnaire. Normalized T-Scores according to the US norms are provided for each subscale with a score of > 60 (> 1 SD from mean) indicating a mildly atypical (possible significant) problem and scores of > 65 indicating a significant problem (markedly atypical) [23]. We used the CRS-R for behavioral assessment of our patients, even though there are no specific norms for children with intellectual disabilities, because no ADHD screening instrument for children with ID is available. The CRS-R reveals adequate psychometric properties with good internal reliability coefficients, high test-retest reliability, and effective discriminatory power [24,25]. The US factor structure was replicated for the German version, as the path relations in the German and US models are 87% identical. Both models show limitations in predictive power [26].

## Results

### Case reports

#### Individual 1

This individual (ES06E1083, subject 1 in Endele et al., 2010 [7]) was born after an uneventful pregnancy at gestational week 41 to healthy and unrelated parents. Family history was uneventful. His birth measurements were within the normal range (Table 1). According to parental account, he showed normal feeding behavior in the newborn period, slept a lot and showed reduced body movements. At two months of age, the parents initially observed muscular hypotonia and a delayed motor development. Physiotherapy was initiated at the age of 6 months. The boy walked without support at 23 months. He spoke his first two words with 12 months, but speech development subsequently stagnated; he spoke about 10 words at the age of 3 years. A developmental test (*Münchener Funktionelle Entwicklungsdiagnostik*, [27]) performed at age 58 months revealed an Intelligence Quotient (IQ) within the range of mild ID. His infection frequency was normal.

Brain MRI at age 17 months was normal. An EEG at the age of 2 years was also normal. At the age of 63 months, left-sided sharp wave complexes were detected; however, seizures were not observed. He carries an apparently balanced *de novo* translocation: 46,XY,t(9;12)(p22;p13.1). The breakpoint in 12p13.1 disrupts the *GRIN2B* gene in exon 4 [7].

Our diagnostic evaluation at the age of 5 ^3^/_12_ years revealed normal body measurements (Table 1). He had a high nasal bridge without any other significant facial anomalies (Figure 1A). He visited a kindergarten for handicapped children and had a delay in fine motor skills and coordination problems; he stumbled and had an unsteady gait. He was able to speak in simple sentences, showed severe delay in receptive speech, and was unable to react properly to verbal demands. He often repeated or imitated sentences spoken by others without understanding the content and without a feeling for the context. He showed primary enuresis diurna and nocturna and encopresis; he was able to eat bite-sized food with a spoon; for hygiene and dressing he totally relied on his parents. He was treated with dipiperone (60 mg in the evening) because of pronounced hyperactivity, increased aggression and major difficulties falling and staying asleep. He woke up every 1–2 hours during the night prior to initiation of the neuroleptic treatment. His attention span did not exceed 10 minutes. He showed temper tantrums, mostly experienced as unpredictable. Aggressive behavior was seen against other children as well as against adults in terms of pinching, biting and hair pulling in a seemingly uncontrolled and undirected manner. Hand flapping and squeaking sounds occurred when he was thrilled. He showed stereotypic and self-injurious behavior such as jumping, shaking or hitting his head and pulling his hair. He preferred strong sensory stimulation like being held tight, pounding and the aforementioned self-injurious behavior. Behavioral problems increased in new or unknown situations that deviated from his daily routine. His understanding of logical associations and consequences was not age-appropriate. He was friendly, sociable and liked to take care of a more severely handicapped child in his school. Social interaction was not impaired. He approached strangers with a trusting and boundless attitude. He acted careless and impulsive in traffic, so that intensive guidance was necessary to avoid accidents.

The behavioral assessment by parents and teachers (DBT, CRS-R:L) was performed at age 6 ^8^/_12._

#### Individual 2

The pregnancy of Individual 2 (ES10E0186, subject 2 in Endele et al., 2010 [7]) was complicated by bleedings. An ultrasound examination revealed a microcephaly at gestational week 32. The boy was born at gestational week 38 to healthy and unrelated parents. Family history was uneventful. Birth measurements were normal except for microcephaly (Table 1). Shortly after birth, a right-sided inguinal hernia, bilateral cryptorchidism and a choanal atresia were surgically corrected. At the age of six months, a bilateral optic atrophy was diagnosed. In his newborn period, he showed normal feeding behavior, but excessive crying during the night. Later in childhood, he was unable to sleep through the night. At age three months, the parents observed that their son did not smile and did not establish eye contact. He walked without support at the age of 3 years.

Brain MRI at the age of 9 months showed hydrocephalus externus with asymmetry of ventricles, potentially due to a prenatal subependymal bleeding. An EEG at the age of 13 months showed irregular slow dysrhythmia and occipital abortive sharp waves, but seizures were not observed. He carries a *de novo* translocation: 46,XY,t(10;12)(q11.23;p13.1), which was assumed to be balanced by conventional karyotyping analysis. However, cloning of the breakpoints revealed that *GRIN2B* is disrupted within exon 2 in 12p13.1; at the breakpoint in 10q21.1 there is an additional *de novo* deletion of 1.1 Mb containing *PRKG1* and *MBL2*.

Upon our diagnostic evaluation at age 12, normal body measurements for height and weight were observed (Table 1), but the microcephaly had persisted. His facial gestalt resembles that of other family members, and no dysmorphic facial features were observed. He visited a school for visually impaired children. He was not able to speak a single word at the age of 12 years, but able to understand simple commands. He was not toilet-trained. He was severely cognitively impaired, friendly but erratic, easily distracted and hyperactive with a short attention span. He preferred strong sensory stimulation, like rocking and loud sounds, and sometimes he hit his head against the ground. His pain perception was reduced. He showed stereotypic behavior like pounding, repetitive movements of his hands and switching the lights on and off. With strangers, he behaved in a trusting manner. Because of his severe ID, he needed intense care and guidance in his daily routine. He was restrained during the night to prevent him from getting up; if left unrestrained he would move around the house and stay awake for prolonged periods of time.

Questionnaires were filled out by the parents at age 14.

#### Individual 3

The boy (ER14077, subject 5 in Endele et al., 2010 [7]) was the first of three children born to healthy non-consanguineous parents. Family history is otherwise unremarkable. Paternal and maternal ages at birth were 29 and 25 years, respectively. Pregnancy and delivery were uncomplicated. Newborn body measurements were in the normal range (Table 1). While no significant abnormalities were noted during the first months of life, delayed achievement of developmental milestones became obvious during the second half of the first year. The boy walked without support at the age of 20 months. According to his parents, first words were spoken around the age of 18 months, but speech remained restricted to single words until the age of 3. The boy has no congenital anomalies, no other physical disorders and no history of seizures (Figure 1B). At age 6 ^6^/_12_, a clinical workup was performed to identify the cause of his developmental delay. It included a metabolic screening, EEG and cranial MRI, which all revealed normal findings. Assessment of cognitive abilities using the Kaufman Assessment Battery for Children [28] at that age showed a homogeneous profile of impairment in all categories with standard values ranging between 54 and 60 (mild ID). He was referred to a genetic workup at the age of 8 ^6^/_12_ years and revisited at the age of 13 years. At age 8 ^6^/_12_, routine genetic testing was negative, including conventional karyotyping, subtelomeric screening by FISH and FMR1 CGG repeat analysis. Physical examination at age 13 revealed no somatic abnormalities. Body measurements were in the normal range (Table 1). The boy carries a missense mutation in the *GRIN2B* gene (c.2044C > T; p.R682C). He showed some impairment in fine motor coordination, but no focal neurologic deficits. He was friendly and cooperative. His language skills were simple for his age, but he could talk in sentences and understand everyday speech.

At the age of 13 years, the boy was attending a special school for intellectually disabled children. He knew almost all letters of the alphabet, was able to read a few words and write his name. He could arrange the numbers from 1 to 20. Teachers described him as friendly with other children. He preferred playing with younger children and liked to play with an elastic strap in his hands. Objects had to stay at their fixed places, he could not handle variations. He was boundless and had close physical contact with others, but also frequent mood swings with episodes of retreat and poor aggression control.

Standardized assessment of behavior with the DBC and the CRS-R:L (filled out by parents and teachers) took place at the age of 14 ^6^/_12._

#### Individual 4

This girl (HDMR179, subject 9 in Endele et al., 2010 [7]) was born following an uneventful pregnancy at 39 weeks of gestation as the second child of non-consanguineous parents. The family history was unremarkable except for Morbus Bechterew in the girl’s mother, the maternal grandfather and a maternal uncle. At the age of 8 months, the girl showed hypotonia and motor delay. She was able to sit at 16 months, walk unsupported at 24 months and climb up stairs at 29 months of age. Her speech development was severely delayed. She spoke her first words at the age of 28 months and the first two-word sentences two months later. At that age, global developmental delay was diagnosed and both speech and motor development corresponded to an age of 13–15 months. Psychological testing with a non-verbal test (Snijders Oomen Non-verbal Intelligence Test Revised, SON-R 2 ½ -7) [29] at the age of 12 years revealed that she had moderate ID and her development corresponded to 3–4 years of age. She was not toilet-trained. EEG was normal. Fragile X syndrome was excluded by molecular analysis of the *FMR1* gene. Chromosome analysis showed a normal 46,XX karyotype at a banding level of 550 bands. FISH analysis did not show a deletion of 17p11.2 (Smith-Magenis syndrome), and subtelomeric imbalances were excluded by MLPA. Molecular karyotyping by Affymetrix 6.0 SNP array did not show copy number variants responsible for her ID and behavioral problems. The girl carries a heterozygous deletion of a dinucleotide in exon 3 of the *GRIN2B* gene (mutation c.803_804delCA, p.T268SfsX15). On diagnostic examination at the age of 13 years, she was friendly but boundless, exhibited excessive talking, but avoided both eye and physical contact. Her body measurements were normal for age (Table 1). She had normal proportions and did not show focal neurological signs. Her appearance resembled that of her mother. Dysmorphic features were absent. Apart from mildly limited extension of the elbows, she did not show further anomalies. She developed behavioral problems at an early age, showing hyperactivity, restlessness, distractibility, echolalia and a very demanding behavior. She liked to switch the lights on and off in a stereotypic manner, and she liked music and loud sounds. She had problems falling asleep and woke up several times a night. She suffered from constipation, but was somatically healthy otherwise.

Questionnaires were filled out by parents and teacher at age 14 ^8^/_12_.

#### Individual 5

The girl (ES10E0097) was the first child born to healthy non-consanguineous parents. Family history was unremarkable. Paternal and maternal ages at birth were 37 and 28 years, respectively. Pregnancy was complicated by bleedings, but delivery was uncomplicated. The girl had normal birth measurements (Table 1). During the first days of life, she presented with myoclonies when falling asleep, but EEG was normal. She had feeding difficulties and cried a lot. She spoke her first words at the age of 10 months and walked without support at the age of 25 months. At the last physical examination, the girl was 23 months old. Her body measurements were normal except for low weight (Table 1). She had no facial dysmorphism or other somatic anomalies (Figure 1C).

After identification of the missense mutation within the *GRIN2B* gene (c.1906G > C; p.A636P), an EEG was performed again. She presented with sharp wave complexes in the temporoparietal region. Generalization to both hemispheres was visible. She had no seizures.

At age 3 ^9^/_12_ years, she showed mild ID. She was able to speak 30–50 single words. She had difficulties falling asleep and awoke once or twice a night. Sometimes she would stay awake for the rest of the night upon awakening. She had a short attention span, was unsettled and needed to move around. She was unable to stay at the table for a whole meal.

Behavioral assessment was conducted at the age of 3 ^10^/_12_ years.

### Results of behavioral assessment

**Figure 2:**
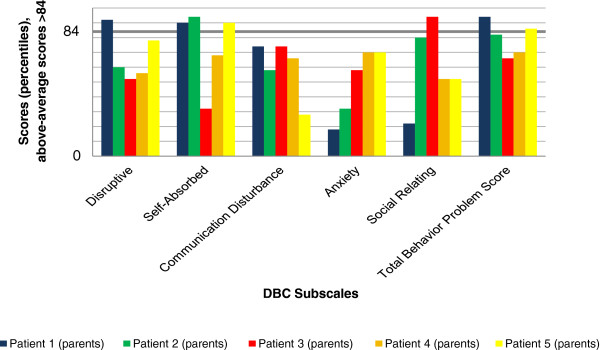
**Parents’ ratings - Developmental Behavior Checklist (DBC).** Above-average scores >84.

**Figure 3:**
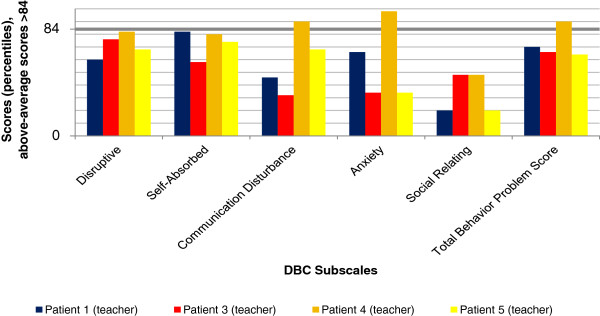
**Teachers’ ratings - Developmental Behavior Checklist (DBC).** Above-average scores >84.

**Figure 4:**
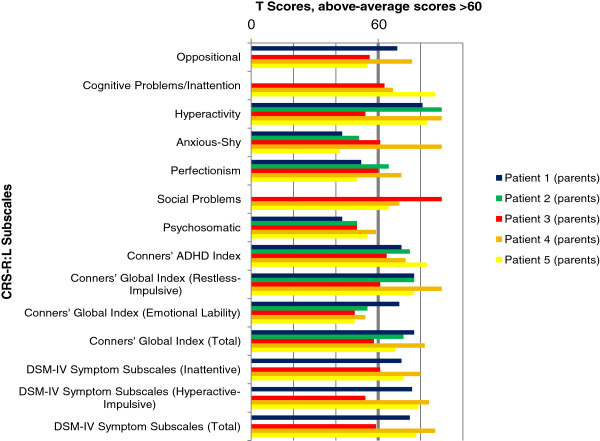
**Parents’ ratings - Conners’ Rating Scales-Revised (CPRS-R:L).** Above-average scores >60.

**Figure 5:**
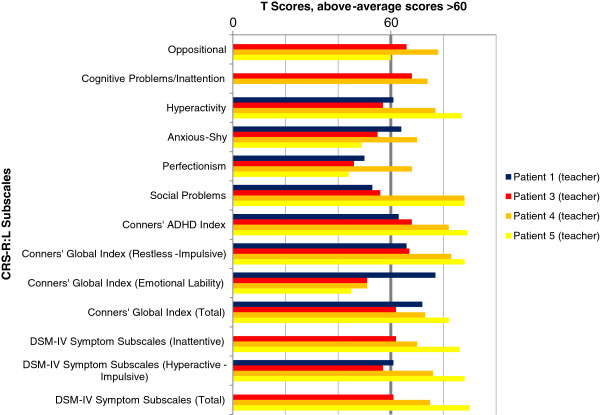
**Teachers’ ratings - Conners’ Rating Scales-Revised (CTRS-R:L).** Above-average scores >60.

The results of parents’ and teachers’ ratings by DBC and CRS-R:L for all five individuals are illustrated in Figures 2, 3, 4, 5, and Tables 2 and 3 show additional data. Teacher’s ratings were not available for Individual 2. Missing data occurred when items of a subscale were not answered.

**Table 2:** Developmental Behavior Checklist (DBC) results (percentiles) for all individuals, rated by parents and teachers

	**Individual 1**	**Individual 1**	**Individual 2**	**Individual 3**	**Individual 3**	**Individual 4**	**Individual 4**	**Individual 5**	**Individual 5**
	**(parents)**	**(teacher)**	**(parents)**	**(parents)**	**(teacher)**	**(parents)**	**(teacher)**	**(parents)**	**(teacher)**
**Disruptive**	92*	60	60	52	76	56	82	78	68
**Self-absorbed**	90*	82	94*	32	58	68	80	90*	74
**Communication disturbance**	74	46	58	74	32	66	90*	28	68
**Anxiety**	18	66	32	58	34	70	98*	70	34
**Social relating**	22	20	80	94*	48	52	48	52	20
**Total problem Behavior sore**	94*	70	82	66	66	70	90*	86*	64

*: above-average scores (>1SD).

**Table 3:** Conners’ rating scales-revised (CRS-R:L) results (T Scores) for all individuals, rated by parents and teachers

	**Individual 1**	**Individual 1**	**Individual 2**	**Individual 3**	**Individual 3**	**Individual 4**	**Individual 4**	**Individual 5**	**Individual 5**
	**(parents)**	**(teacher)**	**(parents)**	**(parents)**	**(teacher)**	**(parents)**	**(teacher)**	**(parents)**	**(teacher)**
**Oppositional**	69*	**x**	**x**	**56**	66*	76*	78*	**55**	**60**
**Cognitive problems/**	x	x	x	63*	68*	67*	74*	87*	x
**Inattention**									
**Hyperactivity**	81*	61*	90*	54	57	90*	77*	83*	87*
**Anxious-shy**	43	64*	51	61*	55	90*	70*	42	49
**Perfectionism**	52	50	65*	60	46	71*	68*	50	44
**Social problems**	x	53	x	90*	56	70*	88*	65*	88*
**Psychosomatic**	43		50	50		59		55	
**Conners’ ADHD index**	71*	63*	75*	64*	68*	73*	82*	83*	89*
**Conners’ global index**	77*	66*	77*	61*	67*	90*	83*	77*	88*
**(Restless-impulsive)**									
**Conners’ global index**	70*	77*	55	49	51	54	51	49	45
**(Emotional lability)**									
**Conners’ global index**	77*	72*	72*	58	62*	82*	73*	68*	82*
**(Total)**									
**DSM-IV symptom subscales (Inattentive)**	71*	x	x	61*	62*	80*	70*	72*	86*
**DSM-IV symptom subscales**	76*	61*	x	54	57	84*	76*	79*	88*
**(Hyperactive-impulsive)**									
**DSM-IV symptom**	75*	x	x	59	61*	87*	75*	78*	90*
**Subscales (Total)**									

*: above-average scores (>1SD); x: missing data.

In summary, all individuals showed considerable behavioral problems. Despite a somewhat inconsistent pattern with respect to the total and subscale scores, it should be noticed that the Conners’ ADHD Index, the Conners’ Global Index (Restless-Impulsive) and the DSM-IV Symptom Subscale (Inattentive) were rated as mildly or markedly atypical for all individuals by parents and teacher, indicating a possible significant problem. The Hyperactivity subscale and the DSM-IV Symptom Subscale (Hyperactive-Impulsive) showed above-average scores for all individuals except Individual 3. The Conners’ Global Index (Total) and the DSM-IV Symptom Subscale (Total) were rated as average only by the teacher of Individual 3. Cognitive Problems/ Inattention were judged as above the normal range in all four children with available data. The Psychosomatic subscale was never rated above average, and Emotional Lability occurred solely in Individual 1.

Results of the DBC were less consistent than those of the CRS-R:L. Self-absorbed behavior was rated as above-average by the parents of Individuals 1, 2 and 5. Disruptive behavior was only rated as clinically relevant by the parents of Individual 1. Communication Disturbance and Anxiety were noticed by the teacher of Individual 4, and Social Relating was mentioned by the parents of Individual 3 alone. The Total Problem Behavior Score revealed marked results for Individual 1 (parents’ judgment), Individual 4 (teacher) and Individual 5 (parents).

Overall, the CRS-R:L revealed higher scores than the DBC. Teachers’ ratings were less often above average than parents’ ratings. This effect was more obvious for the DBC than for the CRS-R:L.

## Discussion

To the best of our knowledge, the present psychological examination of five individuals is the first such study in carriers with mutations in the *GRIN2B* gene. The results are relevant in describing this new behavioral phenotype. Four of five Individuals were reported previously by Endele et al. (2010) [7]. “Behavioral anomalies” were noted in these individuals, but not further described in detail. The objective of our investigation was to provide a detailed description of the behavioral phenotype associated with *GRIN2B* mutations by using clinical assessment as well as standardized questionnaires.

Our five individuals, 2 females and 3 males, aged 3 to 14 years, share some distinctive features: they all show delay in motor and speech development with ID, primary enuresis diurna and nocturna, and encopresis. They all need intense care. Measurements of IQ in our individuals with *GRIN2B* mutation range from mild to severe ID. Individuals 1, 3 and 5 are mildly impaired, Individual 4 shows a moderate ID and Individual 2 is severely retarded, possibly due to an additional *de novo* deletion of 1.1 Mb in 10q21.2 containing the *PRKG1* and *MBL2* genes.

Hyperactivity (4/5), restless-impulsive behavior (5/5), inattention (4/4), oppositional behavior (3/4), and social problems (3/4) stand out as the main behavioral phenotype (Table 1). Individuals 1, 2, 4 and 5 have severe sleeping problems. Stereotypic behaviors with preference for strong stimulation (such as pounding, rocking and loud sounds) and occasional self-injury are frequent. In social interaction, all individuals appear as friendly and sociable, and face others in a boundless and trusting manner. Avoidance of eye contact was described at an early age for Individual 2 and during the current examination of Individual 4, but no further autism-related impairments in interaction and communication were observed.

O’Roak et al. [10] identified a paternally inherited disruptive *GRIN2B* mutation in an individual with evidence of early-onset autism spectrum disorder, possible regression and co-morbidity for mild ID. She had an overall IQ of 63, met the criteria for autism and she was described as hyperactive. This case further confirms our finding that hyperactivity is a major problem in individuals with a *GRIN2B* mutation. In a very recently published paper, the same group reported on *de novo GRIN2B* mutations in four out of 2446 probands with autism spectrum disorder, leading to the assumption that *GRIN2B* belongs to the recurrently mutated genes in ASD/ID phenotypes [17]. No detailed clinical phenotype besides autism was documented in these probands.

The use of standardized questionnaires further contributed to the characterization of the phenotype. Results show marked behavioral problems in all five individuals, also emphasizing hyperactivity and inattention as a clinical hallmark. Self-absorbed behavior is described in three of five individuals. The somewhat inconsistent pattern of problem behavior might partly be due to the individuals’ different ages at diagnostic evaluation (3, 6, and 14 years). Further behavioral assessments in future will clarify if the phenotype with hyperactivity and inattention might become more prominent with higher ages. Regarding missing data, no systematic pattern could be observed. We used the DBC for evaluation of Individual 5 (age 3 ^10^/_12_) despite the questionnaire’s age range from 4 to 18 years, because no assessment instrument for behavioral problems in younger children with ID is available. In Individuals 1 and 4, the behavioral problems had already led to pharmacological treatment, so results of the questionnaires might underestimate deviant behavior. Furthermore, unequal judgments of parents and teachers need to be considered, as they observe the individuals in different contexts. Several studies report low correlations between ratings of parents and teachers [30-32]. Variations between different informants argue for multiaxial assessment of behavior.

The DBC showed less deviant scores than the CRS-R:L. A possible explanation is the use of different reference populations: Standardization of the DBC is based on children and adolescents with ID, thus accounting for findings that children with ID are at an increased risk of developing behavioral/emotional disorders [4-6]. Norms of the CRS-R:L are derived from a population of children without ID. Therefore, above-average scores in this questionnaire might be more readily met and provide more false positives in our five individuals. Nevertheless, we decided to use the CRS-R:L due to the absence of a specific ADHD screening instrument for children with ID. The DBC does not provide a subscale score for inattention or hyperactivity. So, if DBC results are considered exclusively, behavior problems might be underestimated. The comparatively high scores in the CRS-R:L indicate that ADHD symptoms are prominent in *GRIN2B* mutation carriers.

Two of the individuals presented here (Individuals 1 and 2) have a translocation disrupting the *GRIN2B* gene. These two individuals present with the most severe clinical phenotype. One of them (Individual 2) additionally has a *de novo* deletion of 1.1 Mb within 10q21, which might explain the severe ID, additional clinical findings and the progressive microcephaly found exclusively in this individual. However, below-average head circumferences were observed in four of the five individuals, whereas Individual 4 had a circumference of +0.2 SD. Individuals 3 and 5 carry missense *GRIN2B* mutations and have a milder phenotype than the other three individuals. Sharp waves without clinically apparent seizures were diagnosed in individuals 1, 2 and 5, suggesting that this and potentially other EEG abnormalities might occur prominently in mutation carriers. MRI was performed in four individuals with normal findings in three of them. The evaluation of further individuals will help to decide whether these observations are coincidental and to what extent genotype-phenotype correlations exist.

## Conclusion

In conclusion, although specific facial dysmorphism and internal malformations are usually absent in individuals with *GRIN2B* mutations, there does seem to be a characteristic behavioral phenotype consisting of ID, hyperactivity, impulsivity and distractibility. Stereotypic and stimulatory behavior, sleeping problems and a friendly but boundless social behavior also appear to be associated features. EEG anomalies are also helpful to define the phenotype of mutation carriers. Long-term follow-ups are required to determine whether the phenotype becomes more prominent with higher ages.

## Abbreviations

ADHD: Attention deficit/hyperactivity disorder; ASD: Autism spectrum disorder; BMBF: German federal ministry of education and research; CRS-R:L: Conners’ rating scales revised, long version; CPRS-R:L: Conners’ rating scales revised, long version for parents; CTRS-R:L: Conners’ rating scales revised, long version for teachers; DBC: Developmental behavior checklist; DBC-P: Developmental behavior checklist version for parents; DBC-T: Developmental behavior checklist version for teachers; EEG: Electroencephalography; ID: Intellectual disability; IQ: Intelligence quotient; MRD6: Mental retardation, autosomal dominant 6; MRI: Magnetic resonance imaging; MRNET: German mental retardation network; NGFN: National genome research network; NMDA: N-methyl-D-aspartate; SD: Standard deviation.

## Competing interests

The authors declare that they have no competing interests.

## Authors’ contributions

IF carried out the psychological evaluation and drafted the manuscript. BP and SE carried out the molecular genetic studies. RB, UM, HP, E-CP, JR, BZ, MZ, DW investigated the patients and analyzed the clinical data. AR, JH, DW conceived of the study, participated in its design and coordination and helped to draft the manuscript. All authors read and approved the final manuscript.
